# Default Network Deactivations Are Correlated with Psychopathic Personality Traits

**DOI:** 10.1371/journal.pone.0012611

**Published:** 2010-09-07

**Authors:** Tong Sheng, Anahita Gheytanchi, Lisa Aziz-Zadeh

**Affiliations:** 1 Brain and Creativity Institute, University of Southern California, Los Angeles, California, United States of America; 2 Neuroscience Graduate Program, University of Southern California, Los Angeles, California, United States of America; 3 Division of Occupational Science and Occupational Therapy, University of Southern California, Los Angeles, California, United States of America; 4 Pacific Graduate School of Psychology, Palo Alto, California, United States of America; Indiana University, United States of America

## Abstract

**Background:**

The posteromedial cortex (PMC) and medial prefrontal cortex (mPFC) are part of a network of brain regions that has been found to exhibit decreased activity during goal-oriented tasks. This network is thought to support a baseline of brain activity, and is commonly referred to as the “default network”. Although recent reports suggest that the PMC and mPFC are associated with affective, social, and self-referential processes, the relationship between these default network components and personality traits, especially those pertaining to social context, is poorly understood.

**Methodology/Principal Findings:**

In the current investigation, we assessed the relationship between PMC and mPFC deactivations and psychopathic personality traits using fMRI and a self-report measure. We found that PMC deactivations predicted traits related to egocentricity and mPFC deactivations predicted traits related to decision-making.

**Conclusions/Significance:**

These results suggest that the PMC and mPFC are associated with processes involving self-relevancy and affective decision-making, consistent with previous reports. More generally, these findings suggest a link between default network activity and personality traits.

## Introduction

Neuroimaging evidence has identified a network of brain regions that is less metabolically active during goal-oriented tasks than during periods of rest. This network is commonly referred to as the "default network". Because the default network is less active when a task or stimulus is present, it has been argued to underlie non-goal-oriented processes such as the unconstrained recall and maintenance of spontaneous thoughts [1], [2]. While the functions subserved by this network are not completely understood, the network has been suggested to support a baseline state of brain activity [3], and is not likely to reflect task-specific, conscious mental processes [4], [5].

Several brain regions have been identified to be part of the default network, including the posteromedial cortex (PMC), medial prefrontal cortex (mPFC), inferior parietal lobule, and medial and lateral temporal regions [6]. Among these, the midline structures (i.e., the PMC and the mPFC), have been characterized as key component regions of the default network [7]-[9].

The PMC consists of midline parietal structures including parts of the precuneus, posterior cingulate gyrus, and retrosplenial cortex [10], and has been associated with visuospatial integration [11], [12], episodic memory retrieval [13], and affective processes [14], [15]. The mPFC consists of the anterior sectors of the medial frontal gyrus and anterior cingulate gyrus, as well as the non-orbitofrontal sectors of the ventromedial prefrontal cortex. Regions within the mPFC have been associated with decision-making [16]-[18], as well as other affective and social processes [19]-[22]. In addition to their involvement in affective and social processes, both the PMC and mPFC have also been associated with self-referential processes [23]-[26]. This has led to the speculation that the PMC and mPFC may be supporting continuous representations of the self in the absence of tasks [27]-[29].

However, while the PMC and mPFC may play a role in the maintenance of self-related information, the tonically active nature of default network activity suggests that the PMC and mPFC may be involved with more rudimentary processing not specific to particular task-related cognitive functions. In a recent review, Raichle and Snyder described some evidence suggesting that default network activity was unlikely to reflect conscious mental processes [5]. Thus, if the default network indeed supports fundamental background processes such as the integration of internal and external information [3], then individual differences in default network activity might be able to explain differences in personality traits. Evidence supporting this hypothesis comes from several recent neuroimaging studies. Baseline activity in ventromedial prefrontal cortex was found to be correlated with subjects' ratings of negative affect [22]. In addition, regional glucose metabolism in medial prefrontal cortex during rest was observed to correlate with individual differences in neuroticism [30]. These results support the idea that activity in default network regions may underlie aspects of personality and behavioral tendencies.

In the current investigation, we tested the hypothesis that rest-related activity (or task-related deactivations) in default network components, the PMC and mPFC, are correlated with different personality traits related to social, self-referential, and affective processing. We used fMRI to localize the PMC and mPFC and characterized task-related deactivations within these regions of interest (ROIs) using a "Rest - Task" contrast from a speech production task. The task-related deactivations characterized by this contrast (vs. active speech production) involves a period of rest during which no active (motor-related) task demands are present (and, presumably, when some spontaneous background activity may resume), and an instruction to the participant to refrain from producing a motor (speech) action. To assess trait measures related to affective, social, and self-referential processes, we administered the Psychopathic Personality Inventory-Revised (PPI-R) to our participants [31]. We then performed correlation analyses using task-related deactivations within the PMC and mPFC ROIs to determine how strongly each region's deactivations correlated with different trait scores.

Although several personality trait scales have been described previously, we chose the PPI-R because it concentrates on affective and interpersonal components of personality, as opposed to behavioral dimensions. The PPI-R has also been widely used, has been normalized to our participant pool (university students), contains several subscales that relate to the affective, interpersonal, and social dimensions of personality [32], and because psychopathic traits have often been linked to atypical representations of the self. For example, traits such as egocentricity, narcissism, and selfishness are considered components of psychopathy, as individuals who have these traits as dominant aspects of their personality also tend to score higher on psychopathic personality diagnostic measures [33], [34]. Thus, the assessment of psychopathic personality traits might be useful in elucidating how the default network relates to certain personality traits relevant to self, emotion, and social processing. The PPI-R has also been demonstrated to have high reliability estimates [32], [35], and has been validated across different (e.g., clinical and non-clinical, criminal and non-criminal) populations [36], [37].

Based on findings from previous studies that have shown the PMC to be involved in processes related to the self [38], [39], we hypothesized that task-related deactivations in PMC will be correlated with trait scores related to self-absorption (e.g., narcissism, egocentricity). Of the eight subscales on the PPI-R, the Machiavellian Egocentricity (ME) subscale reflects narcissism and egocentric tendencies in a social setting. These trait qualities are closely related to cognitive processes such as self-reflection, processes previously linked to PMC activity. Thus, we predicted that PMC activity during a "rest" condition (task-related deactivation) will be correlated with scores on the ME subscale, as non-task oriented activity of the PMC may related to spontaneous self-referential thoughts or processes.

The mPFC has also been suggested to play a role in aspects of self-referential processing, though its functions may differ from that of the PMC [38]. In addition, because the mPFC has been strongly implicated in aspects of affective and social decision making [16], [19], [40], [41], we hypothesized that task-related deactivations in mPFC will be correlated with traits related to executive processes, such as impulsivity and carelessness. While several subscales of the PPI-R can be interpreted as being related to impulsive behavioral tendencies (e.g., Rebellious Nonconformity (RN), reflecting a "reckless lack of concern regarding social mores" [32]), Carefree Nonplanfulness (CN) reflects an attitude of indifference in planning actions and apparent disregard for consequences [32]. As ventromedial prefrontal patients have been reported to display insensitivity to future consequences (i.e., to delayed reward and punishment feedback) and exhibit behaviors that seem to be driven by immediate feedback [16], [21], the construct represented by the CN subscale seems to closely match descriptions of deficits characteristic of ventromedial prefrontal patients [16]. In contrast, the RN subscale describes a different type of impulsivity centered around anti-authority behavior, and does not typically describe deficits seen in ventromedial prefrontal patients (although it might be relevant to certain cases of orbitofrontal patients, though that is beyond the scope of the current study).

## Materials and Methods

### Participants

Twenty healthy, right-handed, native English speaking volunteers participated in the experiment (13 females, mean age 27.7 years). One participant did not complete the self-report behavioral assessment and was excluded from all analyses. Thus, all subsequent descriptions of the study involved the remaining 19 participants. All participants had normal vision and hearing, and no history of neurological or psychiatric conditions. The protocol was approved by the Institutional Review Board at the University of Southern California, and all volunteers gave written informed consent prior to participating in the study.

### Psychopathic Personality Inventory – Revised

To assess individual differences across multiple psychopathic personality traits, we administered the PPI-R to all nineteen participants. The PPI-R is a self-report measure of global and component traits of psychopathy that measures psychopathic traits along a continuum [32]. The PPI-R consists of 154 items, and participants were asked to rate themselves on how accurately each item described them on a scale of 1 (false) to 4 (true). As the PPI-R is a measure of both global and component traits of psychopathy, it yields a total score (global psychopathy) as well as 8 subscale scores.

Two of the 8 subscales were particularly relevant to the objectives of the current study. The Machiavellian Egocentricity (ME) subscale assesses “narcissistic and ruthless attitudes in interpersonal functioning”, reflecting egocentric tendencies in a social context [31]. A sample item from the ME scale is “I always look out for my own interests before worrying about those of the other guy”. Thus, the ME subscale is closely related to selfishness and self-absorption, psychological processes associated with PMC activity. The Carefree Nonplanfulness (CN) subscale, on the other hand, assesses an “attitude of indifference in planning one's actions”, reflecting a lack of care for the consequences of one's actions [31]. A sample item from the CN scale is “I often make the same errors in judgment over and over again”. The CN subscale is closely related to the type of self-monitoring and affective decision making deficits described in the ventromedial prefrontal patients [16].

### Functional magnetic resonance imaging

All magnetic resonance images were acquired using a Siemens MAGNETOM Trio 3.0 Tesla MRI scanner. Functional volumes were acquired with an echo planar T2*-weighted gradient echo sequence optimal for detecting blood oxygenation level-dependent (BOLD) contrasts (TR = 9000ms; TA = 2000ms; TE = 30ms; flip angle  = 90°; 192mm FoV; 64x64 voxel matrix; 29 axial slices (interleaved); 3x3x4.5mm voxels, no gap). A high-resolution T1-weighted structural scan (MPRAGE; TR = 1950ms; TE = 2.56ms; flip angle  = 90°; 256mm FoV; 256x256 voxel matrix; 208 coronal slices; 1x1x1mm voxels) as well as a T1-weighted structural scan with the same slice prescription as the functional images (coplanar; TR = 702ms; TE = 17ms; flip angle = 55°; FoV = 192mm; 192x192 voxel matrix; 29 axial slices; 1x1x4.5mm voxels) were also acquired from all subjects. Acquisition of functional volumes employed Siemens' prospective acquisition correction (PACE) technique for motion correction, in which head movements are calculated by comparing successively acquired volumes and are corrected online [42].

### fMRI task

The current analyses were performed on data previously collected to investigate prosodic speech production. In that task, which has been previous described in greater detail [43], participants performed a speech production task while being scanned in a sparse sampling event-related paradigm (one functional volume acquired per trial). Each participant completed 3 runs of the task (which were each 7 minutes, 39 seconds in duration). Participants were instructed to produce speech at the offset of a visual cue, according to the cued intonation condition (“happy”, “sad”, “neutral”, “question”). A “rest” condition was also a part of the task, during which a visual cue instructed the participant to produce no speech. Each condition was presented to the subject a total of ten times per run, yielding a total of 50 trials per run. Collapsed across the three runs, thirty trials of each condition were presented to each subject.

In the current investigation, we were interested in whether we would observe task-related deactivations in the PMC and mPFC and whether the two default network components were correlated with different personality traits. We used the contrast “Rest – Speech” (consisting of the subtraction: “rest” condition – all speech conditions; weighted proportionally) to characterize task-related deactivations. This approach provides a contrast estimate of the level of deactivation, and has been described in previous studies [3], [8], [29], [44], [45]. Aside from containing a period when no active task demands were present, our "Rest - Speech" contrast also contained an instruction for the subject to refrain from producing a motor action (speech). The absence of task demands is similar to the rest conditions described in previous reports of task-related deactivations [1], [2], [5], while the inhibitory features could be relevant to the types of executive and cognitive processes that might be related to our specific construct of impulsivity in decision making.

### Data processing and analyses

All images were preprocessed and analyzed with SPM2 software (www.fil.ion.ucl.ac.uk/spm/; Wellcome Department of Imaging Neuroscience, London, UK). Functional images were slice timing corrected, unwarped (from estimated deformation fields), and normalized to the MNI space (using the EPI.mnc template) to allow across-subject comparisons. Images were also spatially smoothed using a 7.5 mm Gaussian filter. Task parameters were estimated using a Finite Impulse Response model (zero filter order, 2 second duration), and motion parameters were calculated from the initial Siemens PACE/MoCo series data and added to the design matrix as a nuisance variable.

### Regions of interest

**Figure 1:**
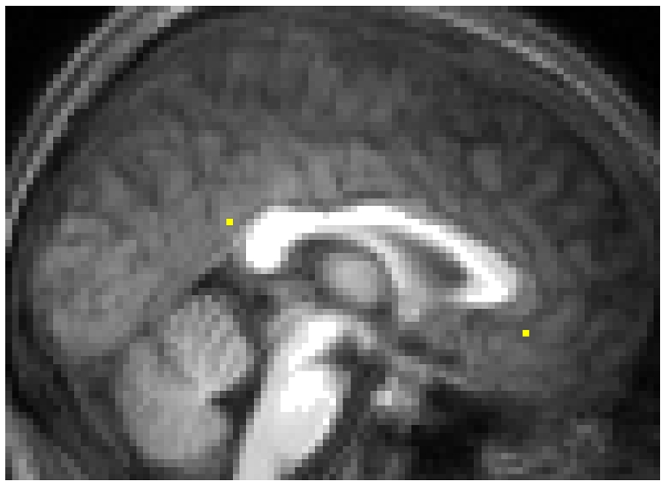
Posteromedial and medial prefrontal regions of interest. The ROIs corresponded to the respective peak voxels of the PMC and mPFC clusters in the contrast (Rest – Speech). The PMC ROI was defined at [0, -48, 24] and the mPFC ROI was defined at [0, 38, -8], in MNI coordinates. ROIs are overlaid onto an averaged anatomical image constructed from the anatomical scans of five representative participants.

Regions of interest (ROIs) were defined for the PMC and mPFC from the “Rest – Speech” contrast at the group level (p<0.05, FDR; T>3.38). Single-voxel ROIs were constructed from the local peak voxels of the PMC and mPFC clusters, respectively. Seed coordinates for the PMC were [0, -48, 24] (all coordinates reported in MNI space), which corresponds to the caudal portion of the posterior cingulate cortex (BA 23&31) [15], [46]. The mPFC seed coordinates were [0, 38, -8], corresponding to BA 32&12. Figure 1 shows the location of the ROIs. Contrast estimates of task-related deactivation were obtained from these ROIs from each participant using MarsBar v0.41 [47], and used in subsequent correlation analyses. Single-voxel ROIs were used (as opposed to FDR-corrected group-level cluster ROIs or spherical ROIs) because we were concerned that the different sizes of the mPFC and PMC clusters may confound results, and that defining a geometric ROI around a peak voxel may also include voxels that were not part of the original cluster. As such, we extracted contrast estimates from only the group peak single-voxel ROIs.

### Correlation analyses

The major focus of the current investigation was to determine whether task-related deactivations in PMC and mPFC were correlated with which aspects of psychopathic personality traits. We expected that the PMC deactivation level would be correlated with the ME subscale of the PPI-R, and that the mPFC deactivation level would be correlated with the CN subscale of the PPI-R. As we were primarily interested in characterizing whether any relationships existed, as well as given our limited sample size, we tested these hypotheses using Spearman's rho, a non-parametric correlation analysis. A *post hoc* correlation analysis describing the overall relationships between PMC, mPFC and all 8 PPI-R subscales was also performed.

## Results

### Correlation analyses

**Figure 2:**
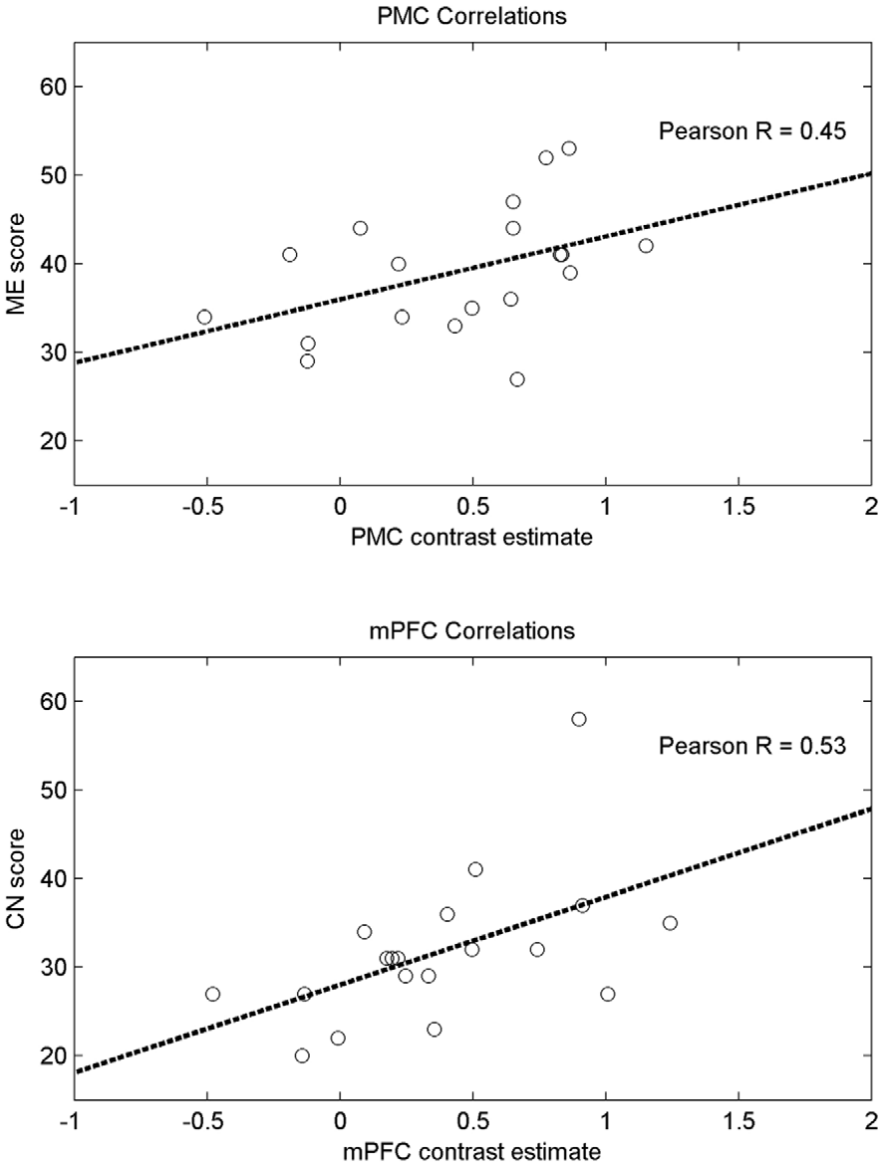
Correlations between task-related deactivations and PPI-R subscale scores. Scatterplots of PMC deactivations and ME scores are shown in the top panel, and scatterplots of mPFC deactivations and CN scores are shown in the bottom panel. Least-squares regression lines are plotted to highlight the relationships between amounts of deactivation and scores on trait scales.

Our results indicate that task-related deactivations in PMC are correlated with ME scores (Spearman's rho  = 0.457; p<0.0495), and that task-related deactivations in mPFC were correlated with CN scores (Spearman's rho  = 0.612; p<0.0053). Table 1 shows the correlation results, and Figure 2 shows scatterplots of the observed relationships. No participants were excluded from analyses as a result of atypical performance on the PPI-R (all participants scored within 2.5 standard deviations of the norm on the embedded Virtuous Responding and Devious Responding validity scales).

**Table 1:** Correlation Matrix of ROIs and PPI-R Measures.

Spearman's rho			
		PMC	mPFC
	PMC		
	mPFC	0.34	
	ME	**0.46** *	0.03
	CN	-0.02	**0.61** *

R-squared			
		PMC	mPFC
	PMC		
	mPFC	0.12	
	ME	**0.21** *	0.00
	CN	0.00	**0.37** *

*p<0.05.

Given the latter results, we were motivated to see if other subscales of the PPI-R would correlate with task-related deactivations in our ROIs. No significant correlations were observed in this correlation analysis (although trends were observed between PMC deactivations and the Blame Externalization (BE; Spearman's rho  = 0.4266) and Stress Immunity (STI; Spearman's rho  = 0.3461) subscales). This correlation matrix is shown in Table 2.

**Table 2:** Correlation Matrix of Default Network ROIs and Other PPI-R Subscales.

Spearman's rho			
		PMC	mPFC
	PMC		
	mPFC	0.3404	
	SOI	0.0483	0.0246
	C	0.2303	0.1964
	F	0.1019	0.0237
	BE	0.4266*	0.0299
	RN	0.1343	0.1088
	STI	0.3461*	0.0200

SOI: Social Infuence.

C: Coldheartedness.

F: Fearlessness.

BE: Blame Externalization.

RN: Rebellious Nonconformity.

STI: Stress Immunity.

No correlations were found at p<0.05. * indicates p<0.1.

## Discussion

Consistent with previous reports on the default network, we found that the PMC and mPFC were less active during a task condition than during a rest condition. Interestingly, deactivation levels within these regions were correlated with individual differences in trait measures of the PPI-R. As predicted, task-related deactivations in PMC were correlated with traits relating to egocentricity, and task-related deactivations in mPFC were correlated with traits relating to decision-making. These results supported our hypothesis that individual differences in task-related deactivations in default network regions might be associated with differences in personality traits.

We found greater levels of task-related deactivation in the PMC in individuals who scored higher on the ME subscale of the PPI-R. This finding suggests that activity during periods of rest in the PMC, a region previously implicated in self-referential processing, is positively correlated with egocentricity and self-absorption. Hence, these regional deactivations may provide a clue to the neural platform for stable self-referential processes associated with narcissism. This result is consistent with previous reports of PMC involvement in the representation of self and self-referential processing [25], [39], [46]. Thus, this finding adds to the growing body of evidence implicating the PMC as a key region supporting the representation of self (though it might do so outside of conscious awareness).

The level of correlation between PMC deactivation and ME scores was rather modest, and this could have been due to several reasons. Although the ME subscale contains a large narcissism/egocentric component, the construct spans a broad range of features, not all of which are relevant to representations of self or self-referential processing. For example, the ME subscale also contains many items involving deceiving and manipulating others and willingness to bend social rules for personal gains [48]. Thus, as the ME construct is so broad, only certain components under its description may be relevant to the types of processes subserved by the PMC.

In addition, we found task-related mPFC deactivation to be correlated with CN scores, with individuals who scored lower on the CN subscale showing less mPFC deactivation. The direction of this correlation may not be intuitive because characteristics such as carelessness and impulsivity have generally been linked to mPFC deficits or impairments, and individuals with less activity in mPFC might be expected to behave more impulsively. However, a positive correlation between mPFC deactivation and CN score would be consistent with the idea that individuals with less mPFC recruitment during active tasks are also more likely to exhibit impulsive traits. Thus, this result reaffirms the involvement of the mPFC in executive processes [17], [18], [21].

Interestingly, although the mPFC has also been implicated in self-referential tasks [9], [49]-[52], mPFC deactivation was not correlated with ME scores. This result was not completely surprising, as the roles of the PMC and mPFC in the representation of self have been suggested to differ [38], [39], [46]. The involvement of the mPFC during self-related tasks could also be that the mPFC was recruited for a general, affective decision-making process present in "self vs. other" discrimination/judgment tasks. This argument would also be consistent with our current understanding of the role of the mPFC in decision making [16], [19], [40], and support our hypothesis that activity in the mPFC should be correlated to measures relating to executive processes.

While we found differential correlation patterns between PMC and mPFC deactivations, we must emphasize that our goal was not to test directly whether PMC and mPFC deactivations correlated differentially with any given personality trait. That is, as we were mainly focused on characterizing whether PMC and mPFC deactivations were associated with different traits, and although the overall correlation patterns observed in Table 1 suggest some degree of functional dissociation between PMC and mPFC, we cannot infer whether, for example, the correlation coefficients for PMC/ME and mPFC/ME were different. Exploratory *post hoc* tests for differences in correlation coefficients revealed no differences between the PMC/ME and mPFC/ME correlations, nor between the PMC/CN and mPFC/CN correlations. This could be due to the fact that, while some functional specialization may be present in the default network, perhaps the PMC and mPFC are engaged in partly overlapping functions. This would be consistent with the literature implicating both cortical midline regions in aspects of self-referential and social processing [15], [19], [38]. Another possibility could be that our experimental design was not powerful enough to detect any differences (and this would be especially true if the processes each region subserves are overlapping). Thus, although characterizing the functional differences between different default network regions has become an area of significant interest, our study may not have been optimally designed to answer this specific question.

Nevertheless, our results seem to be consistent with some recent reports suggesting that cortical midline components of the default network are associated with processes related to self-relevancy and decision-making [53]. In addition, a recent meta-analysis seems to suggest an association between the anterior cingulate/ventromedial prefrontal cortex (anatomically similar to what we labeled mPFC) and features such as action preparation and emotion processing [54].

We should note that our "Rest - Task" contrast differed from those in previous reports in that we used a sparse sampling, event-related design and our "rest" condition contained a visual cue with an instruction to refrain from producing an action. Nevertheless, we reported fairly robust task-related deactivations at the group level. Still, a small number of participants did not demonstrate such deactivations (4 for PMC, 4 for mPFC). As most previous reports of default network deactivations were also based on group-level analyses, the amount and source of variability across subjects are not well understood (although some variance has been attributed to the types of cognitive processes that occur spontaneously during periods of rest). Thus, the current investigation also aimed to contribute to the growing research on characterizing individual differences in default network activity.

Furthermore, our results contribute to the growing body of research that relates rest-related brain activity (in the context of the default network) to personality traits and behavior. While scientists have long been interested in the neural correlates of personality traits [55]-[58], possible links between personality and the default network have only recently been explored [30], [59]. The current investigation show that individual regions within the default network can be related to different personality traits. As the default network has been shown to exhibit intrinsic, spontaneous activity, and suggested to support a baseline level of brain activity, the current results hint at possible relationships between the activity of default network regions (and the rudimentary psychological processes they may subserve) and measurable personality traits and behavioral tendencies.

In addition, these findings demonstrate a novel approach towards elucidating the functions of the default network. An early interpretation of task-related deactivations was that they could reflect inhibition or modulatory processes driven by task demands [60], [61]. However, the consistency with which these task-related deactivations were observed across different studies gave rise to the idea that something much more general was taking place. That the activity profile within these cortical midline structures can explain certain personality traits supports the idea that these brain regions may be involved in general processes such as evaluating environmental and internal signals [3], [5].

As we've focused on two component regions of the default network, our results could be expanded upon through additional research to investigate whether similar interesting results could be obtained if activity throughout the entire default network were examined. We hope future work may provide more insight on the functional specialization of different nodes of the default network, especially with regard to their relationships with personality traits and behavior. In addition, while we administered a self-report inventory (PPI-R) to measure certain psychopathic personality traits, the use of other personality measures to test other specific hypotheses will be invaluable towards understanding the functions of the default network.
